# An Adaptive Control Strategy for DC/DC Converters Using Command-Filtered Backstepping and Disturbance Rejection

**DOI:** 10.3390/mi16121412

**Published:** 2025-12-15

**Authors:** Van Du Phan, Dinh Tu Duong, Van Chuong Le, Sy Phuong Ho

**Affiliations:** School of Engineering and Technology, Vinh University, Nghe An 43100, Vietnam

**Keywords:** buck DC/DC converter, adaptive control, disturbance observer

## Abstract

Ensuring the stability and accuracy of the output voltage in DC/DC buck converters (DBCs) is critical for reliable operation. This paper investigates an observer-based adaptive command-filtered controller designed for DBC systems subject to lumped disturbances. First, a mathematical model of the system is developed on the basis of switching modes. Then, a simplified extended state observer (SESO) is elaborated to mitigate the effects of lumped disturbances. A command filter technique with an integrated adaptive law is subsequently synthesized to enhance output voltage regulation. The stability of the observer and DBC control system is rigorously certified using the Lyapunov principle. Finally, simulation and experimental approaches are exploited to confirm the validity of the proposed method. Compared to state-of-the-art approaches, the proposed observer-based adaptive command-filtered controller improves tracking performance by 96.1% and 77.8% in simulations and 84.4% and 49.1% in experiments under a sinusoidal reference trajectory.

## 1. Introduction

Nowadays, DC/DC buck converters (DBCs) are essential components in power conversion, as they step down input voltages from high to low levels. They find widespread use across multiple applications, including direct current (DC) motor drives [1,2], automotive technology [3], fuel cell systems [4,5], and solar photovoltaic systems [6]. Nonetheless, the unavoidable external disturbances, load variations, high ripple voltages, and uncertainties inherent in nonlinear DBCs pose significant challenges to achieving high-accuracy output voltage tracking regulation [7]. Because of the above issues, various modern control strategies improve the functionality and robustness of DBCs via characteristic merits such as command-filtered backstepping control [8,9], optimal control [10], intelligent control [11,12], and model predictive control [13], along with sliding mode control and its modern version [14,15].

The command filter approach is an advanced form of the backstepping control technique, in which the derivative of the virtual/intermediate control law is computed using a filter [16]. This helps reduce computational complexity. Many applications have adopted this method to leverage its advantages. Well-known examples of fields in which it is used include power electronics [17,18] electro-hydraulic systems [19], and related fields [20,21]. In [22], a Lyapunov function-based backstepping scheme was applied to a practical DBC to increase stability and mitigate the effect of disturbance behavior. In [23], meaningful techniques were introduced including nonlinear state-disturbance estimation and a command filter scheme for high-order general nonlinear systems. In [24], the authors designed an adaptive fuzzy turning-based backstepping sliding mode for power DC/DC converter systems to enhance output voltage performance under external disturbances. As a result, adaptive command-filtered control approaches have become a current research hotspot in advanced control algorithm development.

In practical DC/DC converters, the influence of various perturbations is unavoidable, making it challenging to achieve good dynamic response and high precision. The primary obstacle in controlling DC/DC converters lies in effectively addressing these issues. Many types of observers have been introduced to compensate for such perturbations. In [25], a generalized proportional integral (PI) perturbation observer-based advanced control scheme was constructed for a power DBC to tackle mismatched disturbances and obtain high-precision output voltage performance. In [26], the integration of an extended state observer (ESO) into an extremum-seeking-based adaptive sliding mode control approach for a bidirectional DC-DC converter system was investigated. This approach effectively compensated for external disturbances and achieved high-accuracy output voltage regulation, as demonstrated by simulation results. In [27], a hybrid fuzzy approximation and a non-singular terminal sliding manifold approach were introduced to cope with disturbances and parameter variations affecting a DBC. In [28], the authors synthesized a ESO-based sliding mode technique for DBC systems to attenuate the influence of mismatched disturbances. However, the application of observer-based modern control techniques for DBCs remains limited, and such techniques should be further developed to improve output voltage control performance.

Motivated by the above challenges, this study proposes an observer-based adaptive command-filtered control strategy for DBCs that integrates a simplified extended state observer (SESO) with a command-filtered adaptive control approach. The proposed scheme employs the SESO to proactively compensate for disturbances and estimate system states. The adaptive command filter scheme adjusts the control gains while significantly decreasing computational complexity. As a result, the control system ensures a fast response and precise output voltage tracking. The key contributions of this study may be stated as follows:

(1) It is an innovative attempt to eliminate the influence of a lumped disturbance on a practical DBC by applying an SESO under different voltage reference trajectories.
(2) It involves the design of a new observer-based adaptive command-filtered control strategy for DBCs which delivers excellent performance with a faster response, reduced overshoot, and reduced settling times, compared to other control strategies.
(3) The capabilities and advantages of the proposed control algorithm are demonstrated in both simulation and experimental studies. In addition, the stability of both observer and control system is rigorously verified using the Lyapunov principle.

The remainder of this paper is organized as follows: Section 2 describes the modelling of the output voltage control and the different assumptions for the DBC. Section 3 introduces an SESO design and an adaptive command-filter control strategy, and reports DBC system stability in the presence of lumped disturbance. In Section 4, results obtained from a running simulation and from experiments are investigated to certify the outstanding capability of the proposed control strategy. The conclusion summarizes the main findings of the paper and highlights key areas for future research.

## 2. Dynamic Model of the DBC, and Some Preliminaries

**Figure 1:**
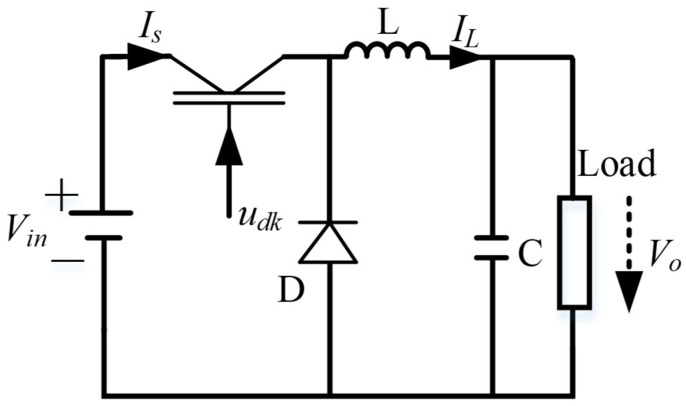
Diagram of the DBC.

The DBC is presented in diagram form in Figure 1. The circuit includes a power supply input, a power switch, a diode, an inductor, a capacitor, and a load. The output voltage of the PEMFC stack using the average model can be calculated as follows [25]:

(1) $$\left\{\begin{array}{c} {\dot{V}}_{o}=\frac{I_{L}}{C}-\frac{V_{o}}{RC}\\ {\dot{I}}_{L}=\frac{uV_{in}}{L}-\frac{V_{o}}{L} \end{array}\right.$$

where *R*, *C*, and *L*, respectively, represent the load resistor, capacitor, and inductor value; *V*_*o*_ denotes the average values of the load resistance output voltage; and *I_L_* defines the current via inductance *L* over a switching frequency. The duty ratio *u* ∈ [0, 1] defines the control signal, and this is the duty cycle of the PWM.

By defining the output voltage *V_o_* as the state variable *x*_1_, and the inductor current *I_L_* as the state variable *x*_2_, the DBC can be determined as follows [29]:

(2) $$\left\{\begin{array}{c} {\dot{x}}_{1}=\frac{1}{C}x_{2}-\frac{1}{RC}x_{1}=f_{1}x_{2}+g_{1}+d_{1}\\ {\dot{x}}_{2}=\frac{V_{i}}{L}u-\frac{1}{L}x_{1}=f_{2}u+g_{2}+d_{2} \end{array}\right.$$

where $f_{1}=\frac{1}{C},f_{2}=\frac{V_{i}}{L},g_{1}=-\frac{1}{RC},g_{2}=-\frac{1}{L},d_{1}=\Delta f_{1}x_{2}+\Delta g_{1},d_{2}=\Delta f_{2}u+\Delta g_{2}$.

$\Delta f_{1},\Delta g_{1},\Delta f_{2},\;\mathrm{and}\;\Delta g_{2}$ denote the uncertainty terms of $f_{1},g_{1},f_{2},\;\mathrm{and}\;g_{2}$. *d*_1_ and *d*_2_ are considered as mismatched and matched disturbances [25].

Assumption 1:

1. The diode and power switch are both ideal. The capacitor is large enough to stabilize the output voltage.
2. The disturbances *d*_1_, and *d*_2_ and their derivatives are bound to satisfy the conditions $\left|d_{i}\right|\leq D_{i}\;\mathrm{and}\;\left|{\dot{d}}_{i}\right|\leq D_{ui},\left(i=1,2\right)$, where $D_{i}\;\mathrm{and}\;D_{ui}$ are positive constants.

## 3. Control Strategy Design

**Figure 2:**
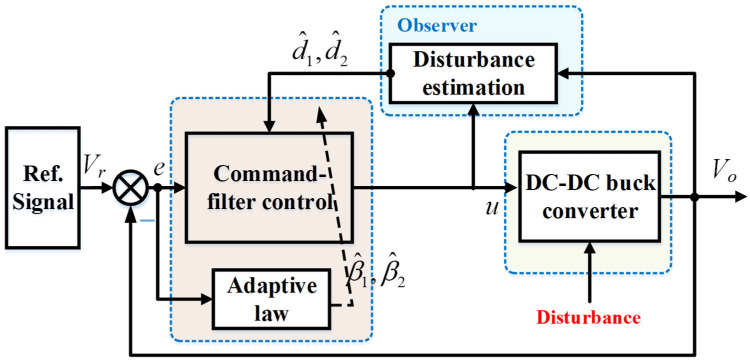
Control structure for DBC with using observer-based adaptive command filter.

In this section, the proposed control algorithm is designed to close the gap between desired voltage and actual output voltage. Disturbance estimation is carried out to first obtain the disturbance information, then compensate for it. An adaptive command-filter control algorithm is then suggested to improve the control performance. The control structure for the DBC is displayed in Figure 2.

### 3.1. Disturbance Estimation Design

The simplified extended state observer (SESO) is constructed as follows:

(3) $$\left\{\begin{array}{c} {\dot{\hat{x}}}_{1}=f_{1}x_{2}+g_{1}+x_{e1}+\alpha_{11}{\tilde{x}}_{1}\;\;\;\;\\ {\dot{\hat{x}}}_{e1}=-\alpha_{12}{\tilde{x}}_{1} \end{array}\right.$$

where *α*_1*i*_ denotes the observer gain (*i* = 1,2), *x_e_*_1_ = *d*_1_ is the extended state, and ${\hat{x}}_{i}\;\mathrm{and}\;{\tilde{x}}_{i}=x_{i}-{\hat{x}}_{i},\left(i=1,2\right)$ define the estimation value and estimation error of the system state *x_i_*. The estimation value of *x_e_*_1_, known as ${\hat{x}}_{e1}$, is computed as follows:

(4) $$\begin{array}{l} {\hat{x}}_{e1}={\dot{\hat{x}}}_{1}-\left(f_{1}x_{2}+g_{1}\right)-\alpha_{11}{\tilde{x}}_{1}\\ ={\dot{x}}_{1}-{\dot{\tilde{x}}}_{1}-\left(f_{1}x_{2}+g_{1}\right)-\alpha_{11}{\tilde{x}}_{1}\\ =d_{1}-{\dot{\tilde{x}}}_{1}-\alpha_{11}{\tilde{x}}_{1}\\ =x_{e1}-\left({\dot{\tilde{x}}}_{1}+\alpha_{11}{\tilde{x}}_{1}\right) \end{array}$$

The observer error can be calculated by ${\tilde{x}}_{e1}=x_{e1}-{\hat{x}}_{e1}={\dot{\tilde{x}}}_{1}+\alpha_{11}{\tilde{x}}_{1}$. Then, the SESO can be re-written as follows:

(5) $$\left\{\begin{array}{c} {\dot{\hat{x}}}_{1}=f_{1}x_{2}+g_{1}+x_{e1}+\alpha_{11}{\tilde{x}}_{1}\\ {\dot{\hat{x}}}_{e1}=-\alpha_{12}{\tilde{x}}_{1}\;\\ {\tilde{x}}_{e1}=x_{e1}-{\hat{x}}_{e1}={\dot{\tilde{x}}}_{1}+\alpha_{11}{\tilde{x}}_{1}\; \end{array}\right.$$

We formulate the SESO for the matched disturbance *d*_2_ as follows:

(6) $$\left\{\begin{array}{c} {\dot{\hat{x}}}_{2}=f_{2}u+g_{2}+x_{e2}+\alpha_{21}{\tilde{x}}_{2}\\ {\dot{\hat{x}}}_{e2}=-\alpha_{22}{\tilde{x}}_{2}\\ {\tilde{x}}_{e2}=x_{e2}-{\hat{x}}_{e2}={\dot{\tilde{x}}}_{2}+\alpha_{21}{\tilde{x}}_{2} \end{array}\right.$$

where *α*_2*i*_ denotes the observer gain (*i* = 1,2), *x_e_*_2_ = *d*_2_ is the extended state, and ${\hat{x}}_{e2}\;\mathrm{and}\;{\tilde{x}}_{e2}$ denote the estimation value and estimation observer error of the matched disturbance *d*_2_.

Observer stability analysis:

Equation (6) can be re-expressed as follows:

(7) $$\begin{array}{l} {\dot{\tilde{x}}}_{e2}={\dot{x}}_{e2}-{\dot{\hat{x}}}_{e2}={\dot{d}}_{2}-\alpha_{22}{\tilde{x}}_{2}\\ {\ddot{\tilde{x}}}_{2}={\dot{\tilde{x}}}_{e2}-\alpha_{21}{\dot{\tilde{x}}}_{2}={\dot{d}}_{2}-\alpha_{22}{\tilde{x}}_{2}-\alpha_{21}{\dot{\tilde{x}}}_{2}\\ \left\{\begin{array}{c} {\dot{\tilde{x}}}_{2}={\tilde{x}}_{e2}-\alpha_{21}{\tilde{x}}_{2}\\ {\ddot{\tilde{x}}}_{2}={\dot{\tilde{x}}}_{e2}-\alpha_{21}{\dot{\tilde{x}}}_{2}={\dot{\tilde{x}}}_{e2}-\alpha_{21}\left({\tilde{x}}_{e2}-\alpha_{21}{\tilde{x}}_{2}\right) \end{array}\right. \end{array}$$

Defining the variable states $z_{1}={\tilde{x}}_{2},z_{2}={\dot{\tilde{x}}}_{2}$, we obtain the following:

(8) $$\left\{\begin{array}{c} {\dot{z}}_{1}=z_{2}\\ {\dot{z}}_{2}={\ddot{z}}_{1}={\dot{d}}_{2}-\alpha_{22}z_{1}-\alpha_{21}{\dot{z}}_{1} \end{array}\right.$$

The characteristic polynomial of the presented system is given by

(9) $$\xi \left(s\right)=s^{2}+as+b,a=\alpha_{21},b=\alpha_{22}$$

From the above selection, $\alpha_{21}=2\mu ,\alpha_{22}=\mu^{2}$, so that Equation (9) becomes $\xi \left(s\right)={\left(s+\mu \right)}^{2}$. Next, it is noted that because the disturbance *d*_2_ is constant, its derivative ${\dot{d}}_{2}=0$. According to Hurwitz’s principle, the original point $\left(z_{1},z_{2}\right)=\left(0,0\right)$ is the stabilizing equilibrium of the system $\xi \left(s\right)={\left(s+\mu \right)}^{2}=0$. Because *d*_2_’s derivative is bounded, i.e., $\left|{\dot{d}}_{2}\right|\leq d_{2m}$, it can be revealed that the observer error is bounded as follows [30]:

(10) $$\left\{\begin{array}{c} {\tilde{x}}_{2}\leq \frac{d_{2m}}{\alpha_{22}}=\frac{d_{2m}}{\mu^{2}}=\mu_{21}\;\;\;\\ {\tilde{d}}_{2}={\tilde{x}}_{e2}\leq \frac{d_{2m}}{\alpha_{21}}=\frac{d_{2m}}{\mu }=\mu_{22} \end{array}\right.$$

Similarly, the convergence of ${\tilde{x}}_{1},\;\mathrm{and}\;{\tilde{x}}_{e1}$ to the bounded region is verified.

**Theorem 1.**  *Considering the system (2) under Assumption 1, there exist positive constants μ_i1_ and μ_i2_ > 0 such that the system error states*${\tilde{x}}_{i},\;\mathrm{and}\;{\tilde{x}}_{ei},i=1,2$ *are bounded, i.e.,*$\left|{\tilde{x}}_{i}\right|\leq \mu_{i1},\;\mathrm{and}\;\left|{\tilde{x}}_{ei}\right|\leq \mu_{i2},i=1,2$*.*

### 3.2. Adaptive Command-Filter Controller

Defining the tracking error states, we obtain the following:

(11) $$\begin{array}{l} e_{1}=x_{1}-x_{1r},\\ e_{2}=x_{2}-x_{2r} \end{array}$$

where *x*_1*r*_ and *x*_2*r*_ define the filtered signals of the intermediate controllers *x*_1*d*_ and *x*_2*d*_. By utilizing a first-order filter, the command filter is established as follows:

(12) $$\rho_{i}{\dot{x}}_{ir}+x_{ir}=x_{id};\;i=1,2$$

where $\rho_{i}>0;x_{1r}\left(0\right)=x_{1d}\left(0\right)$.

The compensated tracking error signals are then given by *ε_i_* = *e_i_* − *y_i_*, with the compensation signal *y_i_* being defined as follows:

(13) $$\left\{\begin{array}{c} {\dot{y}}_{1}=-{\hat{\beta }}_{1}y_{1}+f_{1}\left(y_{2}+x_{1r}-x_{1d}\right)\\ {\dot{y}}_{2}=-{\hat{\beta }}_{2}y_{2}\;\;\;\;\;\;\;\;\;\;\;\;\;\;\;\;\;\;\;\;\;\;\;\;\;\;\;\;\;\;\;\;\;\;\;\;\;\;\;\; \end{array}\right.$$

where *y_i_*(0) = 0, *β_i_* is a nonzero scalar, and ${\hat{\beta }}_{i}$ denotes the estimation value of *β_i_.*

The adaptive law for ${\hat{\beta }}_{i}$ can be given as follows:

(14) $${\dot{\hat{\beta }}}_{i}=\Gamma_{i}\left(\varepsilon_{i}^{2}+\chi_{i}{\tilde{\beta }}_{i}\right),i=1,2$$

where $\Gamma_{i}\;\mathrm{and}\;\chi_{i}>0$.

Taking the derivative of *ε*_1_, the following is yielded:

(15) $$\begin{array}{l} {\dot{\varepsilon }}_{1}={\dot{e}}_{1}-{\dot{y}}_{1}={\dot{x}}_{1}-{\dot{x}}_{1r}+{\hat{\beta }}_{1}y_{1}-f_{1}y_{2}-f_{1}x_{1r}+f_{1}x_{1d}\\ =g_{1}+d_{1}-{\dot{x}}_{1r}+f_{1}x_{1d}+{\hat{\beta }}_{1}y_{1}+f_{1}\left(x_{2}-y_{2}-x_{1r}\right)\\ =g_{1}+d_{1}-{\dot{x}}_{1r}+f_{1}x_{1d}+{\hat{\beta }}_{1}y_{1}+f_{1}\varepsilon_{2} \end{array}$$

The control law *x*_1*d*_ can be given as follows:

(16) $$x_{1d}=\frac{1}{f_{1}}\left(-g_{1}-{\hat{d}}_{1}+{\dot{x}}_{1r}-{\hat{\beta }}_{1}e_{1}\right)$$

The Lyapunov function can be elaborated as follows:

(17) $$V_{1}=\frac{1}{2}\varepsilon_{1}^{2}+\frac{1}{2\Gamma_{1}}{\tilde{\beta }}_{1}^{2}$$

where ${\tilde{\beta }}_{i}=\beta_{i}-{\hat{\beta }}_{i},i=1,2$ denotes the error of the estimated value.

Taking the derivative of *V*_1_, and noting (14) and (16), the following is yielded:

(18) $$\begin{array}{l} {\dot{V}}_{1}=\varepsilon_{1}{\dot{\varepsilon }}_{1}-\Gamma_{1}^{-1}{\tilde{\beta }}_{1}{\dot{\hat{\beta }}}_{1}\\ =\varepsilon_{1}\left(g_{1}+d_{1}-{\dot{x}}_{1r}+f_{1}x_{1d}+{\hat{\beta }}_{1}y_{1}+f_{1}\varepsilon_{2}\right)-\Gamma_{1}^{-1}{\tilde{\beta }}_{1}{\dot{\hat{\beta }}}_{1}\\ =-\beta_{1}\varepsilon_{1}^{2}+\varepsilon_{1}{\tilde{d}}_{1}+f_{1}\varepsilon_{1}\varepsilon_{2}+{\tilde{\beta }}_{1}\varepsilon_{1}^{2}-\Gamma_{1}^{-1}{\tilde{\beta }}_{1}{\dot{\hat{\beta }}}_{1}\\ =-\beta_{1}\varepsilon_{1}^{2}+\varepsilon_{1}{\tilde{d}}_{1}+f_{1}\varepsilon_{1}\varepsilon_{2}-{\tilde{\beta }}_{1}\left(-\varepsilon_{1}^{2}+\Gamma_{1}^{-1}{\dot{\hat{\beta }}}_{1}\right)\\ =-\beta_{1}\varepsilon_{1}^{2}-\chi_{1}{\tilde{\beta }}_{1}^{2}+\varepsilon_{1}{\tilde{d}}_{1}+f_{1}\varepsilon_{1}\varepsilon_{2} \end{array}$$

Applying Young’s inequality, the following is yielded:

(19) $$\varepsilon_{1}{\tilde{d}}_{1}\leq \frac{1}{2}\varepsilon_{1}^{2}+\frac{1}{2}\mu_{12}^{2}$$

${\dot{V}}_{1}$ can now be redesigned as follows:

(20) $${\dot{V}}_{1}=-\left(\beta_{1}-\frac{1}{2}\right)\varepsilon_{1}^{2}-\chi_{1}{\tilde{\beta }}_{1}^{2}+\frac{1}{2}\mu_{12}^{2}+f_{1}\varepsilon_{1}\varepsilon_{2}$$

Taking the derivative of *ε*_2_, the following is yielded:

(21) $$\begin{array}{l} {\dot{\varepsilon }}_{2}={\dot{e}}_{2}-{\dot{y}}_{2}={\dot{x}}_{2}-{\dot{x}}_{2r}+{\hat{\beta }}_{2}y_{2}\\ =f_{2}u+g_{2}+d_{2}-{\dot{x}}_{2r}+{\hat{\beta }}_{2}y_{2} \end{array}$$

The control law *u* can be given as follows:

(22) $$u=\frac{1}{f_{2}}\left(-g_{2}-{\hat{d}}_{2}+{\dot{x}}_{2r}-{\hat{\beta }}_{2}e_{2}-f_{1}\varepsilon_{1}\right)$$

The Lyapunov function can be designed as follows:

(23) $$V_{2}=V_{1}+\frac{1}{2}\varepsilon_{2}^{2}+\frac{1}{2\Gamma_{2}}{\tilde{\beta }}_{2}^{2}$$

Taking the derivative of *V*_2_, and noting (18) and (22), the following is yielded:

(24) $$\begin{array}{l} {\dot{V}}_{2}={\dot{V}}_{1}+\varepsilon_{2}{\dot{\varepsilon }}_{2}-\Gamma_{2}^{-1}{\tilde{\beta }}_{2}{\dot{\hat{\beta }}}_{2}\\ ={\dot{V}}_{1}+\varepsilon_{2}\left(f_{2}u+g_{2}+d_{2}-{\dot{x}}_{2r}+{\hat{\beta }}_{2}y_{2}\right)-\Gamma_{2}^{-1}{\tilde{\beta }}_{2}{\dot{\hat{\beta }}}_{2}\\ =-\left(\beta_{1}-\frac{1}{2}\right)\varepsilon_{1}^{2}+\frac{1}{2}\mu_{12}^{2}+f_{1}\varepsilon_{1}\varepsilon_{2}+\varepsilon_{2}\left({\tilde{d}}_{2}-{\hat{\beta }}_{2}\varepsilon_{2}-f_{1}\varepsilon_{1}\right)-\Gamma_{2}^{-1}{\tilde{\beta }}_{2}{\dot{\hat{\beta }}}_{2}\\ =-\beta_{1}\varepsilon_{1}^{2}+\varepsilon_{1}{\tilde{d}}_{1}+f_{1}\varepsilon_{1}\varepsilon_{2}-\beta_{2}\varepsilon_{2}^{2}-{\tilde{\beta }}_{2}\left(-\varepsilon_{2}^{2}+\Gamma_{2}^{-1}{\dot{\hat{\beta }}}_{2}\right) \end{array}$$

Applying Young’s inequality, the following is yielded:

(25) $$\varepsilon_{2}{\tilde{d}}_{2}\leq \frac{1}{2}\varepsilon_{2}^{2}+\frac{1}{2}\mu_{22}^{2}$$

Substituting (14) and (25) into (24), ${\dot{V}}_{2}$ can be represented as follows:

(26) $${\dot{V}}_{2}\leq -\sum_{i=1}^{2}\left(\beta_{i}-\frac{1}{2}\right)\varepsilon_{i}^{2}-\sum_{i=1}^{2}\chi_{i}{\tilde{\beta }}_{i}^{2}+\sum_{i=1}^{2}\frac{1}{2}\mu_{i2}^{2}$$

**Theorem 2.**  *Considering the system (2) under assumption 1, if the control law is designed as in (16) and (22), and the SESO is designed as in (5) and (6), all system states *$\varepsilon_{i}\;\mathrm{and}\;{\tilde{\beta }}_{i}$ *are bounded and converge to a small region in the presence of lumped disturbances.*

**Proof.**  Select the Lyapunov candidate function *V* = *V*_2_. From (26), $\dot{V}$ can be written as follows:

(27) $$\dot{V}\leq -HV+M$$

where $H=\min\left(\lambda_{\min}\left(\beta_{i}-\frac{1}{2}\right),\lambda_{\min}\left(\chi_{i}\right)\right),M=\sum_{i=1}^{2}\frac{1}{2}\mu_{i2}^{2}$. □

From further analysis of (27), the following is obtained:

(28) $$V\leq \left(V\left(0\right)-\frac{M}{H}\right)e^{-Ht}+\frac{M}{H}\leq \Lambda ,$$

where $\Lambda =V\left(0\right)+\frac{M}{H}.$

From (28), lim_t⟶∞_*V* = $\frac{M}{H}$, and it can be revealed that the variables $\varepsilon_{i}\;\mathrm{and}\;{\tilde{\beta }}_{i}$ are uniformly bounded.

Therefore, Theorem 2 is completed.

## 4. Application Verification

### 4.1. Numerical Simulation

In this part, simulation studies are carried out using MATLAB/Simulink(Version 2024a). The frequency is set as 5 kHz. The simulation setup parameters of the DBC are as follows: *R* = 30 Ω; *L* = 0.01 H; *C* = 0.01 F. The output voltage of the DBC is required to vary from 0 V to 12 V with an input voltage of *V*_in_ = 24 V. In this paper, the load variations (e.g., a 10% RC load change) and system parameter deviations (e.g., a 15% change in inductance) are treated as components of the lumped disturbance. In detail, the lumped disturbances are expressed as $d_{1}=0.1\times \left(f_{1}x_{2}+g_{1}\right),d_{2}=0.15\times \left(f_{2}u+g_{2}\right)$. The initial conditions are $x_{1}\left(0\right)=0,x_{2}\left(0\right)=0$. To examine the superior performance of the suggested algorithm (S3), other control strategies are considered: an adaptive command-filtered control strategy (S1) and an observer-based command-filtered backstepping control strategy (S2).

The control gains of the proposed control strategy (S3) are chosen as follows: *μ* = 30; Γ_1_ = Γ_2_ = 0.2; χ_1_ = χ_2_ = 0.3; *β*_1_ = 130; *β*_2_= 45; *ρ*_1_ = *ρ*_2_ = 0.01. The adaptive command-filtered control strategy (S1) differs from S3 as it does not incorporate disturbance estimation. The observer-based command-filtered backstepping control strategy (S2) differs from S3 in that it does not include the adaptive law. In both cases, all other parameters are kept identical to those of S3.

**Remark 1.**  *Increasing β_1_, β_2_, and μ, and decreasing Γ can effectively reduce the tracking, disturbance, and adaptation errors. Nonetheless, selecting values for these parameters beyond appropriate bounds may impair the overall performance of the DC/DC system, manifesting as overshoot or chattering. Therefore, it is imperative to determine suitable control parameters that align with the operating characteristics of the controlled system.*

#### 4.1.1. Case Study 1

**Figure 3:**
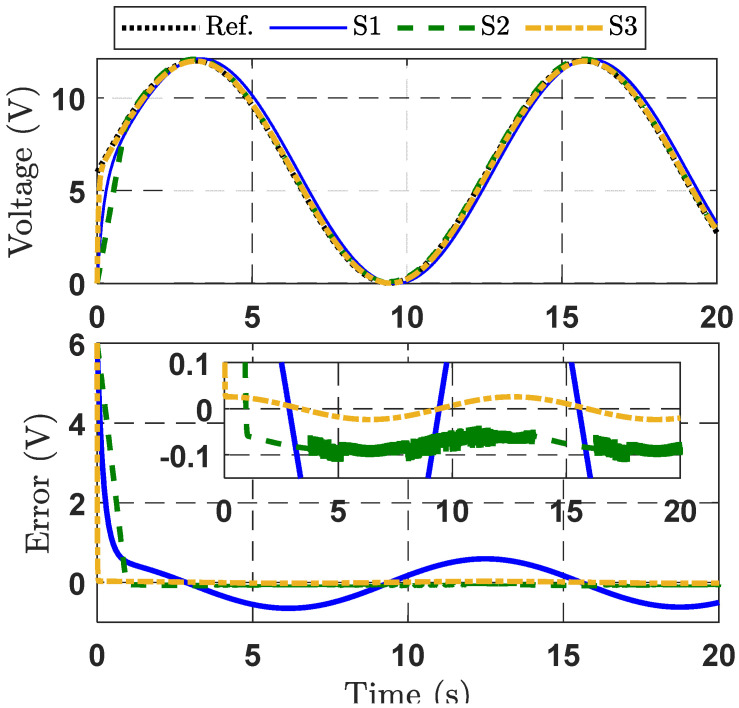
Response curves for the three control strategies, Case Study 1.

In this case study, the desired output voltage trajectory is given by $x_{1d}=6+6\sin\left(0.5t\right)\left(V\right)$ to evaluate the results of three control strategies. Tracking performance with respect to output voltage control is plotted in Figure 3. It is obvious that the proposed control strategy (S3) yields a faster response in reaching the set point than the other control strategies (S1 and S2). Furthermore, we can evidently see through the zoomed part in the second subgraph of Figure 3 that the output tracking voltage error of S1 is the largest because of the lack of disturbance compensation. The S2 strategy reduces the tracking error, but it remains greater than under the proposed control strategy. It is apparent from these figures that the suggested controller provides superior output voltage tracking performance in comparison with the other control strategies.

#### 4.1.2. Case Study 2

**Figure 4:**
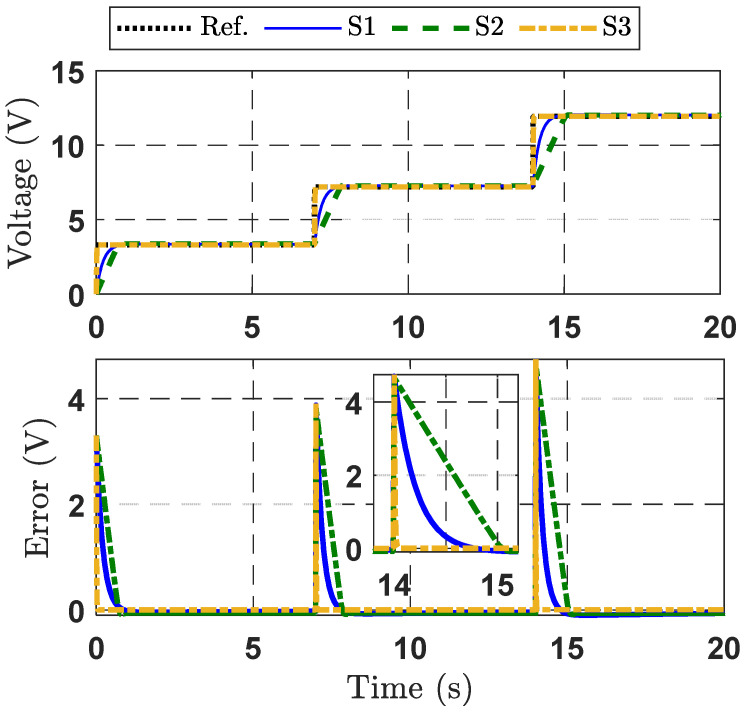
Response curves for the three control strategies, Case Study 2.

In this case study, to further evaluate the control performance of three control strategies, the desired output voltage trajectory is given by $x_{1d}=3.3\left(V\right)\;\mathrm{with}\;0\leq t\leq 7,x_{1d}=7.2\left(V\right)\;\mathrm{with}\;7\leq t\leq 14,x_{1d}=12\left(V\right)\;\mathrm{with}\;14\leq t\leq 20$. The tracking performances of the three strategies with respect to output voltage control, including output voltage tracking and tracking error, are shown in Figure 4. The first subgraph of Figure 4 illustrates that the output voltage of the DBC effectively tracks the reference trajectory. In particular, the proposed control strategy (S1) outperforms the other strategies (S2 and S3). The second subgraph of Figure 4 presents the output voltage tracking error. In the zoomed-in view from 13.5 s to 15.5 s, the tracking error of the proposed control strategy converges to zero within 0.02 s. This is significantly faster than S2 and S3, both of which converge after approximately 1 s. As observed, strategy S1 performs better than strategies S2 and S3 in the presence of lumped disturbances. This superior performance is due to S1 being designed by combining the advantages of the SESO and the adaptive command filter technique. In contrast, the absence of disturbance compensation or an adaptive law leads to poorer control performance in S2 and S3. These results demonstrate the key performance benefits of the proposed controller.

To further appraise the quality of the presented control strategies, two performance indices—maximum error (MAXE) and root mean square error (RME) [31] are provided, as shown in Table 1. These values are calculated over the duration of the last 16 s. The indicator values for the S1 strategy are reduced in comparison to the other strategies (S2 and S3), confirming the superior effectiveness of S1.

### 4.2. Experimental Results

**Figure 5:**
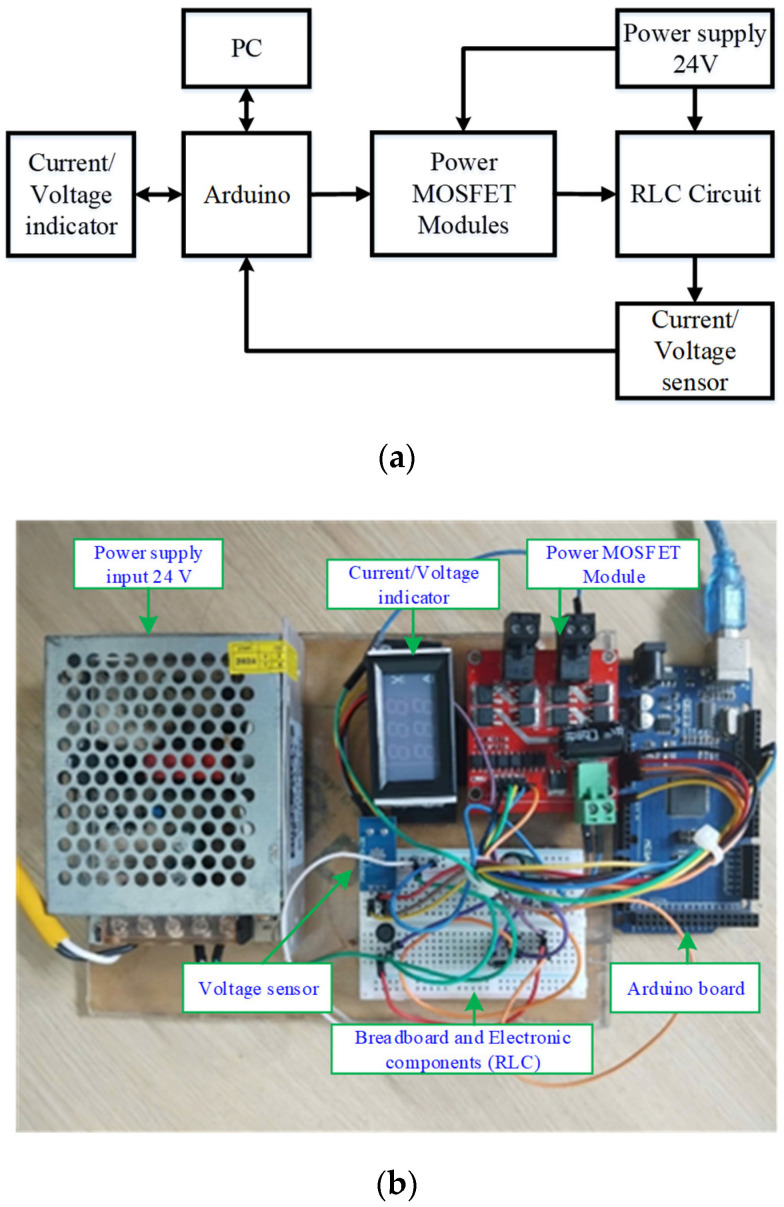
DBC system test bench. (**a**) Schematic diagram of the setup. (**b**) Main DBC circuit.

In this subsection, the observer-based adaptive command-filtered controller designed for DBC systems under lumped disturbances is validated on a real-time system through several experiments. The experimental DBC test bench is configured as shown in Figure 5. Figure 5a illustrates the wiring connections of the hardware components, while Figure 5b shows the complete DBC system. The setup includes a 24 V power supply, a current sensor, a voltage sensor, an RLC circuit, integrated PWM and power MOSFET modules, a microprocessor connected to a PC, an electronic test board, and a monitor. As illustrated in the schematic diagram in Figure 5a, MATLAB/Simulink is executed on the laptop PC, and the observer-based adaptive command-filter controller is developed via a MATLAB function. The generated control signal is sent to PWM pin 5 of the Arduino, which subsequently supplies the input to the Power MOSFET module. Accordingly, the embedded control platform comprises the laptop PC running MATLAB/Simulink, the Arduino, and the Power MOSFET module.

The main aim of the experiments is to demonstrate the practical feasibility of the suggested control approach for output voltage tracking in the DBC. The same two scenarios as in the simulation are conducted as a sinusoidal and multistep reference trajectory. During the experiment, the control gains of the proposed control strategy (S3) are re-examined and determined as follows: *μ* = 40; Γ_1_ = Γ_2_ = 0.15; χ_1_ = χ_2_ = 0.3; *β*_1_ = 150; *β*_2_= 50; *ρ*_1_ = *ρ*_2_ = 0.02.

#### 4.2.1. Experimental Case Study 1

**Figure 6:**
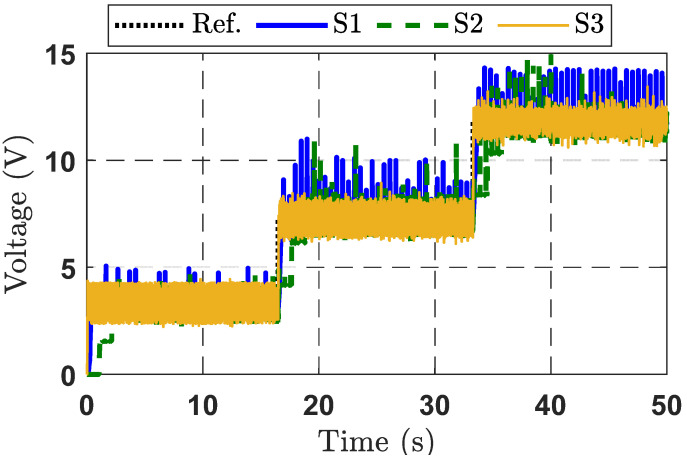
Output voltage response curves for the three control strategies in Experimental Case Study 1.

Output voltage tracking performance in Experimental Case Study 1 is shown in Figure 6. It is noteworthy that the suggested control methodology (S3) results in less output voltage fluctuation compared to the other strategies (S1 and S2). It may be observed that the brown line (S3) exhibits the smallest overshoot and the shortest rise time.

**Figure 7:**
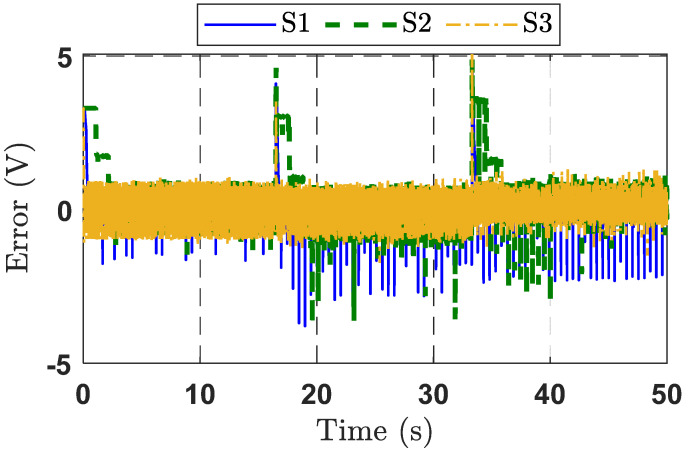
Tracking error response curves for the three control strategies in Experimental Case Study 1.

**Figure 8:**
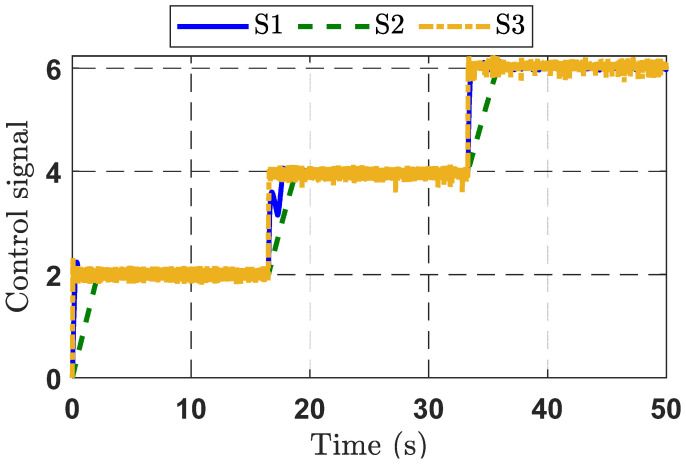
Control signal response curves for the three control strategies in Experimental Case Study 1.

Output voltage tracking errors are presented in Figure 7. The tracking error of the proposed control strategy is significantly reduced thanks to the integration of disturbance compensation and the adaptive law for adjusting control gains. The S2 strategy shows the poorest control performance, particularly at 17 s and 34 s where the desired signal experiences a voltage step. Overall, the analysis confirms that the S3 strategy provides the best output voltage tracking performance, compared to the other control strategies. The control signals of the three control strategies are depicted in Figure 8.

#### 4.2.2. Experimental Case Study 2

**Figure 9:**
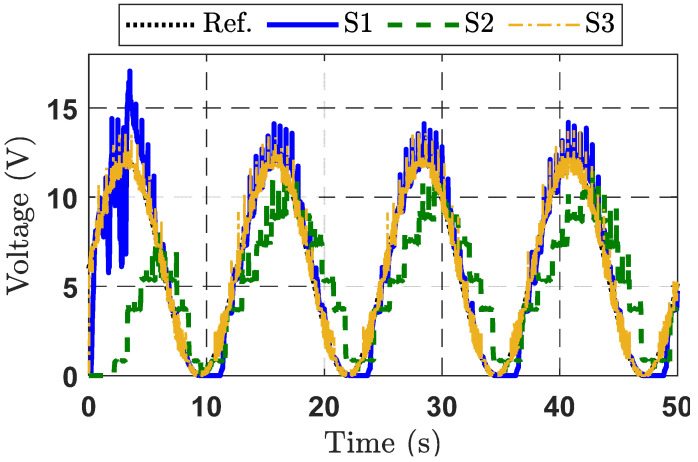
Output voltage response curves for the three control strategies in Experimental Case Study 2.

**Figure 10:**
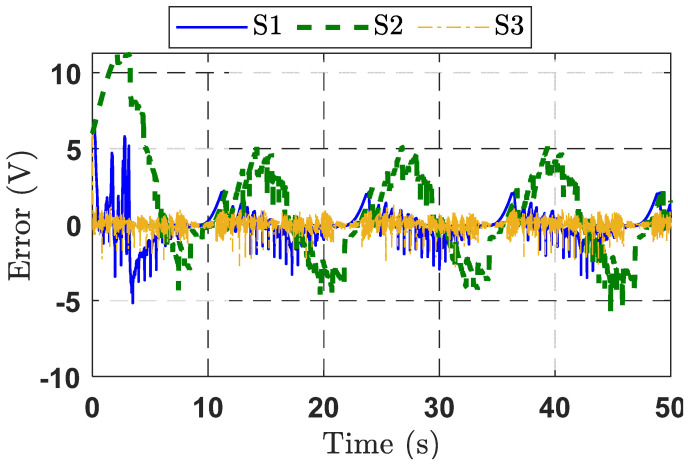
Tracking error response curves for the three control strategies in Experimental Case Study 2.

**Figure 11:**
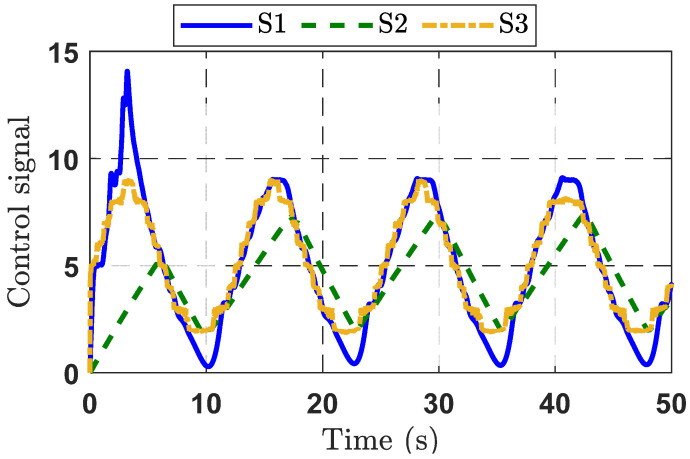
Control signal response curves for the three control strategies in Experimental Case Study 2.

To further validate the output voltage control capability of the observer-based adaptive command-filtered controller, the desired sinusoidal signal was applied to a DBC system. The results of this second experiment are displayed in Figure 9, Figure 10 and Figure 11.

From Figure 9, the responses of the three strategies (S1, S2, and S3) indicate that the proposed control strategy S3 delivers better performance compared to S1 and S2. The S2 strategy fails to track the target signal effectively. Although the S1 strategy improves the rise time, it exhibits noticeable overshoot. Furthermore, from the tracking error response curves for the three control strategies presented in Figure 10, it can be seen that the S1 strategy achieves the smallest error, thanks to the integration of the observer and the adaptive command filter technique. The control signal response characteristics of the three control methods are depicted in Figure 11.

Table 2 presents a quantitative performance analysis demonstrating the benefits of the proposed control strategy. It is worth noting from Table 2 that the indicators MAXE and RME for the S3 strategy are lower than those for the other methods (S1 and S2).

The performance measures obtained in both the simulation and experimental studies clearly demonstrate that the proposed observer-based adaptive command-filtered controller outperforms the other control strategies in all respects. These findings verify the practicality and feasibility of applying the proposed algorithm to real-world DBCs.

## 5. Conclusions

In this paper, a robust command-filtered control strategy is designed to enhance the output voltage control performance of a DBC. A lumped disturbance that affects output performance is addressed using a simplified extended state observer (SESO). The proposed SESO not only estimates the disturbance but also the system states. Furthermore, a command filter is introduced to eliminate complex deviations. The combination of the SESO and command filter improves the output voltage tracking, bringing it closer to the desired signal. Numerical simulations and various experimental studies were conducted to demonstrate the superiority of the proposed control algorithm. Tracking performance improved by 96.1% and 77.8% in simulations and by 84.4% and 49.1% in experiments under a sinusoidal reference trajectory. Therefore, the suggested control algorithm is effective in boosting efficiency and compensating for the effects of lumped disturbances. In future work, experimental studies on fault-tolerant control of the DBC will be investigated.

## Figures and Tables

**Table 1:** Performance indicators for the three control strategies in the simulation.

	Control Strategy	MAXE (V)	RME (V)
Case Study 1	S1	0.6537	0.4653
	S2	0.1157	0.0812
	S3	0.0259	0.0180
Case Study 2	S1	4.6953	0.4966
	S2	4.7414	0.8507
	S3	4.6501	0.1574

**Table 2:** Performance indicators for the three control strategies in the experimental case studies.

	Control Strategy	MAXE (V)	RME (V)
Case Study 1	S1	5.5052	1.0141
	S2	5.0552	0.5229
	S3	4.8843	0.4633
Case Study 2	S1	3.4966	0.7852
	S2	5.7634	2.5688
	S3	2.8034	0.4003

## Data Availability

Data are contained within the article.
